# Supplementary material for: Patterns of Intron Gain and Loss in Fungi
Source: PLoS Biol. 2004 Nov 30;2(12):e422. doi: 10.1371/journal.pbio.0020422 (PMC532390; doi:10.1371/journal.pbio.0020422)
Supplement: Table S1 — Also available at http://genes.mit.edu/NielsenEtAl/. (4.3 MB ZIP). [file pbio.0020422.st001.zip › NielsenEtAl/html/67.html]

AN8050.1.NCU09013.1.MG01675.1.FG06908.1


```
 CLUSTAL W (1.82) Multiple Sequence Alignments - Introns Inserted


Sequence 1: MG01675.1	146 aa
Sequence 2: FG06908.1	188 aa
Sequence 3: NCU09013.1	148 aa
Sequence 4: AN8050.1	173 aa
Alignment Length: 217 aa
Number Identitical Residues: 99 aa
Alignment Score (without introns) 4362


MG01675.1 	------~------------------------------------MSWFQKQFTLPAKSRGS
NCU09013.1	------~------------------------------------MSWSQKTFTLPAQSRGA
FG06908.1 	MPHYDG1LFHFIWTLPFTLLVFSGSSQPRPTALPVIDHSTATSMSWFQKQFTLPAKSRGS
AN8050.1  	------~------------------------------------MPWTQKTFTLPSKSRGS
          	                                           *.* ** ****::***:

MG01675.1 	YLITDTVVKEIPEIKNYKVGLLNLFVQHTSCALSLNENW~DE~DVREDMSNALDRIAPAA
NCU09013.1	YLITDIVTKEVPEIKQYKVGLLNLFIQHTSCALSLNENW~DP0DVRLDMSDTLDRIVPEA
FG06908.1 	YLITDQVVSSLPEIRDFKVGLLNLFVQHTSCALSLNENW1DS~DVREDMSDALDRIAPAE
AN8050.1  	YLITDHILSSLPEIREYKVGMLNLFVQHTSCALSLNENW~DE~DVREDMSDALDRIAPAD
          	***** : ..:***:::***:****:************* *  *** ***::****.*  

MG01675.1 	GPKGESLYLHS~AEGPDDMP0AHIKSALVGASVTIPISNGKL~ATGTWQGIW2YLEFR2A
NCU09013.1	TSKNPNLYRHN~DEGPDDFP~AHVKSSLIGTSVTIPITNGKL0ATGTWQGIW~YLEFR~A
FG06908.1 	GPKGEALYRHD2AEGPDDMP~AHIKSALIGASVTIPIKDGKL0ATGTWQGIW~YLEFR~A
AN8050.1  	--KKGNLYRHS~AEGEDDMP0AHIKSALIGASVNIPISNGKL~ATGTWQGIW~YLEFR~A
          	  *   ** *.  ** **:* **:**:*:*:**.***.:*** ********* ***** *

MG01675.1 	SKHQRKIVATIQGEKM~--------~---------------------
NCU09013.1	HKHRRNIVATIQGEKG~KE------~---------------------
FG06908.1 	ARHQRRVVATIQGEKA~--------~---------------------
AN8050.1  	MRHTRKVVATIQGEKD2IRVLLLVV1APSVTRWFSFTSSAAPVGSRG
          	 :* *.:********           :.: :   : :::::. .: .
```
